# Medicina traslacional y fibrilación auricular: ¿qué nuevas terapias tenemos disponibles?

**DOI:** 10.47487/apcyccv.v6i2.484

**Published:** 2025-06-27

**Authors:** Juan Santiago Serna-Trejos, Stefanya Geraldine Bermúdez-Moyano, Carlos Andrés Castro-Galvis

**Affiliations:** 1 Universidad ICESI, Cali, Colombia. Universidad Icesi Universidad ICESI Cali Colombia; 2 Unidad de Cuidado Intensivo, Hospital Universitario del Valle, Cali, Colombia. Unidad de Cuidado Intensivo Hospital Universitario del Valle Cali Colombia; 3 Pontificia Universidad Javeriana Cali, Cali, Colombia. Pontificia Universidad Javeriana Pontificia Universidad Javeriana Cali Cali Colombia

*Sr. Editor*

La fibrilación auricular (FA) es la arritmia sostenida más frecuente a nivel mundial, con una prevalencia estimada de más de 50 millones de casos en 2020 y una proyección superior a los 12 millones, solo en Estados Unidos para 2030. Su incidencia y carga global se encuentran en ascenso, impulsadas por el envejecimiento poblacional, el aumento de la obesidad, la hipertensión y la mejor detección clínica. Desde una perspectiva epidemiológica, la FA se asocia con un riesgo 2,4 veces mayor de accidente cerebrovascular, cinco veces mayor de insuficiencia cardíaca y un exceso de mortalidad del 50 al 100%, lo que la convierte en un importante problema de salud pública con impacto clínico, económico y pronóstico sustancial ^1^.

La fisiopatología de la FA implica la interacción entre disparadores ectópicos, usualmente localizados en las venas pulmonares, y un sustrato auricular vulnerable caracterizado por remodelación eléctrica, estructural y anatómica. La activación anómala por fugas de calcio en el retículo sarcoplásmico y la desregulación de canales iónicos promueven despolarizaciones tempranas y actividad ectópica, mientras que el acoplamiento celular deficiente, la fibrosis intersticial y la heterogeneidad en el período refractario efectivo facilitan mecanismos de reentrada. Estos procesos, modulados por factores como la edad, la obesidad, la hipertensión y la insuficiencia cardíaca, configuran una miocardiopatía auricular progresiva que perpetúa la FA y explica su curso clínico heterogéneo ^2^.

Diversos estudios han confirmado la implicación de variantes genéticas en la fisiopatología de la FA, especialmente aquellas que afectan canales iónicos y proteínas de acoplamiento celular. Mutaciones en genes de canales de potasio (como KCNQ1, KCNA5, KCNN3) y sodio (SCN5A, SCN1B, SCN10A) pueden alterar la repolarización auricular y favorecer mecanismos de reentrada o actividad ectópica. Asimismo, mutaciones en genes como GJA5 (conexina 40) y NUP155 (nucleoporina) han sido asociadas con formas familiares de FA de inicio temprano. Estudios de asociación genómica amplia (GWAS) han identificado loci relevantes, como PITX2, PRRX1, CAV1 y ZFHX3, los cuales modulan procesos de desarrollo auricular, acoplamiento eléctrico y remodelación estructural. Además, se ha observado que polimorfismos en micro-ARNs, como miR-21 y miR-133, regulan la expresión génica relacionada con fibrosis e inestabilidad eléctrica, lo que refuerza la complejidad del componente hereditario en la FA ^3^.

Por lo anteriormente descrito, teniendo en cuenta el sustrato genético de la FA, surgen novedosas investigaciones en el marco de futuras terapias emergentes para el manejo de esta condición. Tal es el caso de Wiedmann *et al*., quienes evaluaron el inhibidor del canal de potasio activado por calcio KCa2, AP30663, como un nuevo agente antiarrítmico en un ensayo clínico de fase 2 en pacientes con FA reciente. Este fármaco mostró una tasa de conversión a ritmo sinusal del 55% con la dosis de 5 mg/kg y del 42% con 3 mg/kg, frente a 0% con placebo. La probabilidad bayesiana de superioridad fue >99,9% para ambos brazos activos, superando el umbral preespecificado. La ausencia de arritmias ventriculares y un perfil de seguridad comparable al placebo refuerzan su selectividad auricular. AP30663 se posiciona, así como un prometedor candidato para el control de ritmo en FA sin el riesgo de proarritmia ventricular que limita los antiarrítmicos convencionales ^4^.

Paasche *et al.* demostraron que la dapagliflozina, un inhibidor de SGLT2, ejerce efectos antiarrítmicos agudos mediante la modulación directa de la excitabilidad de los cardiomiocitos auriculares. En modelos celulares y animales, redujo significativamente la velocidad de ascenso del potencial de acción y la densidad de corriente de sodio pico, efectos que favorecieron la reversión de FA paroxística y el control del ritmo en FA persistente. Estos hallazgos extienden el espectro terapéutico de los SGLT2i más allá de la protección cardiorrenal, sugiriendo un rol emergente como agentes clase I funcionales para el manejo agudo de FA, con potencial aplicación clínica, especialmente en el contexto de insuficiencia cardíaca ^5^.

Wiedmann *et al*. exploraron el reposicionamiento del fármaco doxapram como inhibidor del canal TASK-1, un canal de potasio específico de la aurícula cuya sobreexpresión en FA contribuye al acortamiento del potencial de acción. En un modelo porcino de FA persistente, el doxapram redujo de forma significativa la carga de FA, restauró la duración del potencial de acción y mostró eficacia tanto para cardioversión como para mantenimiento del ritmo sinusal. Los experimentos en cardiomiocitos humanos confirmaron estos efectos. Estos resultados respaldan su evaluación clínica en el ensayo DOCTOS y consolidan al canal TASK-1 como un blanco terapéutico altamente específico y clínicamente viable ^6^.

Bai *et al.* propusieron a la sulcardina (Sul), un derivado de la changrolina, como agente antiarrítmico multicanal que bloquea las corrientes de sodio (INa, IC₅₀=26.9 μmol/L) y calcio tipo L (ICa,L, IC₅₀=69,2 μmol/L) sin afectar las corrientes de potasio rectificadoras. En modelos animales, la Sul aumentó de manera dosis-dependiente el umbral de arritmias inducidas por la ouabaína y prolongó el período refractario efectivo. A diferencia de los antiarrítmicos previos, la Sul no mostró un potencial proarrítmico significativo, posiblemente debido a su acción balanceada sobre múltiples canales iónicos, posicionándose como un candidato interesante para el tratamiento de FA sin compromiso estructural ventricular significativo ^7^^)Sul^.

A pesar de los avances recientes, el tratamiento farmacológico de la fibrilación auricular continúa limitado por la baja especificidad de las terapias disponibles, su eficacia transitoria y el riesgo persistente de efectos adversos graves. Esta realidad evidencia la necesidad de replantear la manera en que se desarrollan los agentes antiarrítmicos, incorporando modelos que consideren la heterogeneidad estructural, eléctrica y genética de la FA. La medicina traslacional, al integrar la caracterización molecular con datos funcionales y clínicos, se posiciona como el eje fundamental para diseñar estrategias terapéuticas más seguras y dirigidas. En este contexto, emergen múltiples blancos terapéuticos con potencial para redefinir el control de ritmo en FA (Figura 1), cuya validación en estudios clínicos será clave para su incorporación efectiva en la práctica cardiovascular.

**Figura 1:**
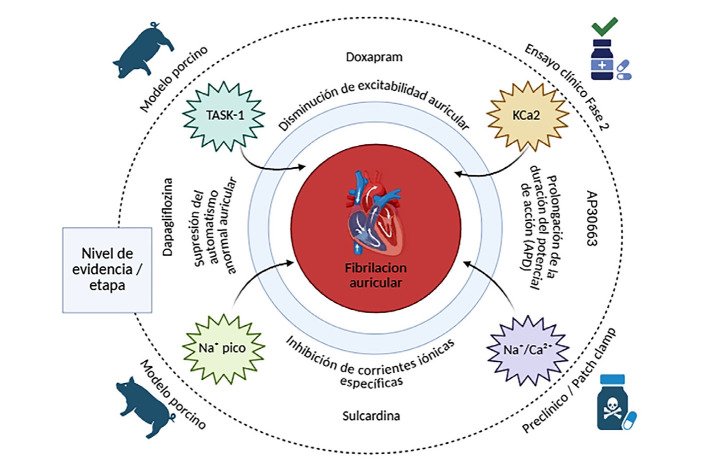
Nuevos blancos terapéuticos en fibrilación auricular: una visión traslacional

## Financiamiento:

Autofinanciado
